# Modeling habitat connectivity in support of multiobjective species movement: An application to amphibian habitat systems

**DOI:** 10.1371/journal.pcbi.1008540

**Published:** 2020-12-28

**Authors:** Timothy C. Matisziw, Ashkan Gholamialam, Kathleen M. Trauth

**Affiliations:** 1 Department of Geography, University of Missouri, Columbia, Missouri, United States of America; 2 Department of Civil & Environmental Engineering, University of Missouri, Columbia, Missouri, United States of America; 3 Institute for Data Science and Informatics, University of Missouri, Columbia, Missouri, United States of America; University of Illinois at Urbana-Champaign, UNITED STATES

## Abstract

Reasoning about the factors underlying habitat connectivity and the inter-habitat movement of species is essential to many areas of biological inquiry. In order to better describe and understand the ways in which the landscape may support species movement, an increasing amount of research has focused on identification of paths or corridors that may be important in providing connectivity among habitat. The least-cost path problem has proven to be an instrumental analytical tool in this sense. A complicating aspect of such path identification methods is how to best reconcile and integrate the array of criteria or objectives that species may consider in traversal of a landscape. In cases where habitat connectivity is thought to be influenced or guided by multiple objectives, numerous solutions to least-cost path problems can exist, representing tradeoffs between the objectives. In practice though, identification of these solutions can be very challenging and as such, only a small proportion of them are typically examined leading to a weak characterization of habitat connectivity. To address this computational challenge, a multiobjective optimization framework is proposed. A generalizable multiobjective least-cost path model is first detailed. A non-inferior set estimation (MONISE) algorithm for identifying supported efficient solutions to the multiobjective least-cost path model is then described. However, it is well known that unsupported efficient solutions (which are equally important) can also exist, but are typically ignored given that they are more difficult to identify. Thus, to enable the identification of the full set of efficient solutions (supported and unsupported) to the multiobjective model, a multi-criteria labeling algorithm is then proposed. The developed framework is applied to assess different conceptualizations of habitat connectivity supporting amphibian movement in a wetland system. The results highlight the range of tradeoffs in characterizations of connectivity that can exist when multiple objectives are thought to contribute to movement decisions and that the number of unsupported efficient solutions (which are typically ignored) can vastly outweigh that of the supported efficient solutions.

## Author summary

Biological studies and experiments have provided many insights as to the complex nature of the criteria (objectives) that species may evaluate in decisions regarding inter-habitat landscape traversal. Those insights are often used to develop models for identifying paths or corridors potentially supporting inter-habitat movement to characterize habitat connectivity from the species’ perspective. However, typically only a small proportion of the alternative paths that may also support species movement and be important to habitat connectivity are identified. Thus, the computational challenge is to develop methods that can more completely characterize the set of paths/corridors that may support habitat connectivity. To address this challenge, a modeling framework that better integrates the objectives thought to influence inter-habitat movement is outlined, facilitating the identification of a broader set of paths reflecting the tradeoffs among the objectives. Through an application of the developed framework to model habitat connectivity for multiobjective amphibian movement in a wetland system, it is demonstrated that an extensive and diverse set of efficient inter-habitat paths can be identified. The capability to characterize these additional dimensions of habitat connectivity supporting species movement can provide researchers and practitioners with a means to develop more robust representations of complex biological systems.

This is a *PLOS Computational Biology* Methods paper.

## Introduction

Research has widely reported changes in species persistence over the past 30 years [1,2]. Urbanization, infrastructure, and habitat transformation are frequently cited as among the leading factors responsible for these changes [3,4]. Given the rapid pace of environmental and landscape change, it is important to understand the factors and mechanisms that may influence habitat connectivity to address management and conservation concerns [5]. For example, preserving or creating inter-habitat corridors that best meet the needs of species for dispersal events (e.g., natal dispersal) as well as part their regular migration (e.g., mating, foraging, and summer-winter habitat) is critical to the persistence of species, especially in human-dominated landscapes [6,7].

### Landscape connectivity and conservation of biodiversity

The extent to which the landscape supports species movement among habitats is often referred to as landscape or habitat connectivity [8,9]. Connectivity in this sense is a complex function of landscape and species-specific characteristics. As such, a wide array of metrics for quantifying connectivity have been proposed, many of which are rooted in network theory given the need to link the spatial structure of complex systems to prospects for movement therein [10,11]. In network models of landscape systems, habitat areas (i.e., patches) are represented as nodes and the direct linkages between the habitat nodes are represented as arcs. Connectivity between a pair of habitat areas can therefore be modeled as the set of arcs a species traverses en route from one habitat to another, often termed a path or corridor. In networked systems though, a multitude of paths between a pair of nodes may exist. Therefore, decisions need to be made as to which paths are actually viable alternatives capable of supporting a particular type of movement. For example, a common assumption in modeling movement is that travel in a system involves costs and hence, efficient movements with respect to those costs are more desirable. A frequently utilized measure of connectivity among habitats in this respect is the shortest or least-cost path [12,13]. In this sense, the relative cost of movement associated with traversing arcs connecting landscape features for a particular species is quantified based upon how different landscape and ecological factors are thought to impede or facilitate movement [14]. Once the cost of traversing arcs in the landscape system has been established, the most efficient inter-habitat path(s) is then often sought as a measure of habitat connectivity [12]. That is, it is assumed that paths (or corridors) that have the lowest cumulative cost (i.e., resistance or impedance) from a species’ perspective are more likely to be important in supporting inter-habitat movements [15,16].

### Modeling ecological networks – least-cost paths

The mathematical model used for identifying paths of minimal costs in a network is known as the shortest path problem or more generally as the least-cost path problem. Methodologically, least-cost path problems involve a network *G* with *N* nodes and *A* arcs, *G*(*N*,*A*) in which a path between an origin node (*o*∈*N*) and a destination node (*d*∈*N*) is sought. In least-cost path problems, the decisions are to identify whether or not each arc (*i*,*j*)∈*A* should be included as part of the path. These decisions are typically modeled using binary-integer variables *x*_*ij*_ = {0,1} ∀(*i*,*j*)∈*A*, where *x*_*ij*_ = 1 if an arc (*i*,*j*) is selected as part of the path and *x*_*ij*_ = 0, otherwise.

The objective (or criterion) to be optimized in least-cost path problems is usually some function of the arc decision variables (*x*_*ij*_) and their associated costs (*c*_*ij*_), such as the product of the arc cost and associated decision variable as in Eq (1) [17]. Feasible solutions to a least-cost path problem are those that adhere to Constraints (2)-(3).

$$Minimize\;\Omega =\sum_{(i,j)\in A}c_{ij}x_{ij}$$ (1)

s.t.

$$\sum_{j|(i,j)\in A}x_{ij}-\sum_{j|(j,i)\in A}x_{ji}=\left\{ \begin{array}{l} 1\;\mathrm{for}\;i=o\\ 0\;\forall i,i\neq o,d\\ ‐1\;\mathrm{for}\;i=d \end{array}\right.$$ (2)

$$x_{ij}=\left\{0,1\right\}\;\forall (i,j)\in A$$ (3)

More specifically, Constraints (2) are conservation of flow conditions and ensure that: a) an arc that exits the origin node is selected, b) an arc that enters the destination node is selected, and c) for all nodes other than the origin and destination, if a selected arc enters a node, an arc that exits the node must also be selected. Constraints (3) stipulate that all arc decision variables are binary-integer, though it is known that relaxing the binary-integer restriction (0≤*x*_*ij*_≤1) will also result in a binary-integer solution [17]. Exact solutions to many forms of least-cost path problems can be readily obtained using well-known algorithms, such as that of Dijkstra [18]. Given that these types of algorithms are not computationally burdensome and are very accessible, they have been widely implemented in open-source and commercial software products [19,20] and are commonly applied in ecological research.

In order to derive a least-cost path, the cost of moving among habitats (i.e., nodes) through the intervening landscape (i.e., the arcs) must first be quantified. In many ecological applications, the landscape is partitioned into a set of analysis areas (e.g., raster cells or polygons). Each area is assigned a cost reflecting the relative resistance it presents to movement. The cost of traversing an analysis area is usually derived based on the assumed contribution of different landscape characteristics present within the area. For example, combinations of landscape characteristics such as forest canopy, land use and land cover, habitat quality, elevation, road density and proximity to water are frequently used in deriving landscape traversal cost [12,21–23]. After each analysis area has been assigned a cost, a network can be constructed in which the nodes represent the analysis areas to be traversed with the arcs representing the spatial connection between neighboring areas. The arcs can then be attributed with the cost values from the corresponding analysis areas and the least-cost path model can be applied, yielding a single optimal path between an origin and destination node.

While least-cost paths based on composite measures of cost have been widely explored, such cost representations have been viewed as lacking a robust biological or empirical foundation [13]. While there is evidence that species utilize some sort of decision-making framework when navigating the landscape, the exact nature of the framework and the combination of factors upon which it is premised has not been well established. For example, there are many objectives that have been postulated regarding the amphibian decision-making processes in seeking new habitat, such as: minimizing distance, minimizing elevation change, maximizing exposure to moist environments, and maximizing likelihood of successful traversal [24–27]. Further, the exact combination(s) of objectives that may underlie movement decisions is unknown.

Measurement of the objectives can also present challenges as even small differences in how costs are quantified for arcs can influence the location and characteristics of the resulting least-cost paths [28]. Given that the cost of traversing arcs is often derived based on a combination of factors, the way in which the factors are combined can be a major source of uncertainty in the representation of an ecological system [15]. Further, the fact that there are essentially an infinite number of ways in which costs representing different objectives can be weighted and combined perhaps remains one of the most challenging obstacles to application of multiobjective least-cost path problems and interpretation thereof. As is described next, a myriad of Pareto-optimal or efficient solutions can exist for a multiobjective least-cost path problem. However, the efficient solutions that are usually identified in practice, likely represent only a very small sample of those that exist given the solution methodologies that are commonly employed. Measures of habitat connectivity premised upon a limited set of solutions are therefore also likely to only represent weak estimates of connectivity.

### Multiobjective optimization

Multiobjective approaches serve to integrate a broader set of criteria into analysis/planning problems. Unlike with single objective optimization models (e.g., the least-cost path problem), in multiobjective models, there can be many solutions, each optimal with respect to some mix of the objectives considered (termed Pareto-optimal solutions). Eq (4) is a generic multiobjective least-cost path problem in which there are a set of *l*∈*L* objectives. Each objective *l* represents some function of the arc decision variables and their associated cost components $\left(c_{ij}^{l}\right)$. The multiple objectives are subject to Constraints (2) and (3) as in the single objective least-cost path problem.

$$Minimize\;\left(\left(f_{1}(c_{ij}^{1}x_{ij})|(i,j)\in A\right),\ldots ,\left(f_{\left|L\right|}(c_{ij}^{\left|L\right|}x_{ij})|(i,j)\in A\right)\right)$$ (4)

Consider a set of *feasible* solutions (those that do not violate the constraints) *S* to a multiobjective optimization problem. Given a feasible solution *s*∈*S*, if there is no other feasible solution *s*∈*S* in which $f_{l}(c_{ij}^{l}x_{ij}^{s})\leq f_{l}(c_{ij}^{l}x_{ij}^{s^{*}})$, s* is considered to be an *efficient* or *Pareto-optimal* solution and the corresponding Pareto-frontier $f_{l}(c_{ij}^{l}x_{ij}^{s^{*}})$ is termed *non-dominated* [29]. Thus, in the full set of Pareto-optimal solutions S*, each solution is better than (efficient) all others with respect to at least one criterion.

Within the set of efficient solutions, some exist on the convex boundary of the solution space. These solutions are termed *supported* and can be found by techniques such as the weighting method, NISE, and MONISE. In the weighting method, the objectives are combined into a single minimization problem in which each objective *l*∈*L* is assigned a weight (*w*_*i*_), such that *w*_1_+*w*_2_,…,+*w*_|*L*|_ = 1.0 [30]. For example, given two objectives Ω_1_ and Ω_2_, one weighting scheme might be to set both objective weights to 0.5 (e.g., *Minimize* 0.5Ω_1_+0.5Ω_2_). The resulting model has the same form as the single-objective least cost path problem and can therefore be solved as such. Once solved, the result is a single supported efficient solution and associated non-dominated path. In order to identify other efficient supported solutions, different combinations of weights can be applied and the resulting models solved in order to search for other supported efficient solutions to approximate the supported efficient set. For example, another weighting scheme might involve weighting one objective by 0.8 and the other by 0.2 (e.g., *Minimize* 0.8Ω_1_+0.2Ω_2_). This is by far the most common approach for addressing multi-criteria least-cost paths in ecological studies [12,15,31–33]. Although the weighting method is straightforward to apply, its utility for identifying all supported efficient solutions is typically very limited. In cases in which two objective are to be optimized, the non-inferior set estimation (NISE) method can be applied to estimate the set of efficient solutions [34]. This process involves evaluating the solution space between pairs of supported efficient solutions to detect the presence of another supported efficient solution. When new supported efficient solutions are found, the solution space between them and their neighboring supporting solutions is in turn evaluated for the presence of additional supported efficient solutions. Therefore, NISE provides a means for identifying all supported efficient solutions in biobjective optimization problems. In the case that more than two objectives are to be considered, the NISE approach becomes more complicated [35,36]. In order to cope with these complexities, multiple objective non-inferior set estimation (MONISE) techniques have been proposed to extend the NISE concept to characterize the supported efficient frontier when more than two criteria are involved [37–39].

While solution techniques such as the weighting method, NISE and MONISE can assist with providing an estimate of the efficient set (the supported efficient solutions), other efficient solutions can also exist between supported solutions along non-convex portions of the solution space. These *unsupported* efficient solutions are more challenging to identify, but can represent sizable portions of the Pareto-optimal solution set, the number of which can increase exponentially with the size of the optimization problem [40]. The complete set of efficient solutions (supported and unsupported) to a multiobjective least-cost path model can be identified using a class of solution algorithms known as exact multi-criteria labeling algorithms [41,42]. Multi-criteria labeling algorithms start by first examining an origin node, iteratively visiting neighboring nodes, and assigning labels representing traversal cost for the tentative paths connecting the origin node to other nodes. Every time a new non-dominated path is found, a node’s label is updated, and this process continues until all nodes are labeled, at which point all efficient (supported and unsupported) paths between the origin and destination node are found. While labeling algorithms are very effective solution methods, they can be applied only if the path cost is separable among its component arcs and if the monotonicity of the cost functions can be guaranteed [43]. In cases where those conditions cannot be satisfied, a subset of the efficient solutions can be heuristically identified by imposing a threshold constraint on one of the objectives, enumerating all paths that meet the threshold constraint, and then applying a filtering technique to retrieve those that are efficient [44].

While the applicability of multiobjective least-cost path approaches to biological and ecological problems has been demonstrated in literature, the tendency has been to utilize solution methods that yield a limited number of supported efficient solutions. As such, there are likely many other valid, important, solutions to these problems that are not being evaluated and analyzed that could provide fruitful insights. The other supported and unsupported efficient solutions to multiobjective least-cost path problems can provide more insight on the nuanced tradeoffs between the characteristics of the paths potentially supporting habitat connectivity for a species. In particular, consideration of the unsupported efficient solutions is especially important given the fact that they can often constitute a major proportion the solutions in the efficient set. To this end, a general multiobjective framework for modeling paths/corridors supporting habitat connectivity is described. A MONISE algorithm is then detailed for identifying supported efficient solutions to the multiobjective problem. An exact multi-criteria labeling approach for identifying all efficient solutions (supported and unsupported) to the multiobjective problem is then described. An application of the multiobjective model (and solution techniques) to connectivity in amphibian habitat systems is then provided to highlight the utility of the proposed approach.

## Materials and methods

### Multiobjective habitat connectivity problem

A multiobjective *habitat connectivity* problem (MOHCP) is proposed for accounting for a general set of objectives that could be modeled in a least-cost path framework. In particular, three objectives assumed to influence the inter-habitat movement of a species are integrated in the model: a) minimize the total risk associated with movement [9,45], b) minimize the total distance traveled [22,46], and c) minimize change in environmental conditions encountered during movement [26,27]. To model these objectives, each arc (*i*,*j*) in the network is associated with attributes reflective of environmental change (*z*_*ij*_), travel distance (*c*_*ij*_), and risk associated with landscape traversal (*π*_*ij*_). For each origin-destination (*o*,*d*∈*N*) habitat pair in the network, the MOHCP can be formulated as follows:

$$Minimize\;\Omega_{1}^{od}=1-\Pi_{(i,j)\in A}{\left(1-\pi_{ij}\right)}^{x_{ij}}$$ (5)

$$Minimize\;\Omega_{2}^{od}=\sum_{(i,j)\in A}c_{ij}x_{ij}$$ (6)

$$Minimize\;\Omega_{3}^{od}=\sum_{(i,j)\in A}z_{ij}x_{ij}$$ (7)

s.t. (2) & (3)

Objective (5) minimizes the risk (*π*_*ij*_ = [0,1]) of traversal failure. This objective is analogous to maximizing the likelihood of successful traversal. Objective (6) minimizes the total distance traveled. Objective (7) minimizes the total change in environmental conditions encountered. Constraints (2) and (3) are applied as in the regular least-cost path problem.

Given that the probability of successful traversal of each arc is (1−*π*_*ij*_) = [0,1], Objective (5) is monotonically increasing, a sufficient condition for Bellman’s principal of optimality [43]. Thus, all sub-paths of a Pareto-optimal path with respect to Objective (5) are also Pareto-optimal. Objectives (6) and (7) are also monotonic, therefore, all sub-paths of Pareto-optimal solutions with respect to these objectives are Pareto-optimal as well. While Objective (5) is nonlinear and non-additive, it can be re-stated in an additive and linear form by modifying the log transformation function proposed by Reinhardt and Pisinger [47] as in Eq (8).

$$Minimize\;\Omega_{1}^{od}=\sum_{(i,j)\in A}x_{ij}\ln\;(\frac{1}{1‐\pi_{ij}})$$ (8)

### Solution methodologies

As discussed earlier, the weighting method is commonly used to identify some of the supported efficient solutions to problems like the MOHCP. However, the extent to which those supported efficient solutions represent the complete set of efficient solutions cannot be determined. Thus, alternative methods for characterizing the efficient set should be explored. In this spirit, a MONISE routine is described for identifying the set of supported efficient solutions and an exact multi-criteria labeling routine is detailed for identifying the complete set of efficient solutions to the MOHCP (Eqs (5)–(7) & (2)-(3)).

#### MONISE for MOHCP

**Fig 1:**
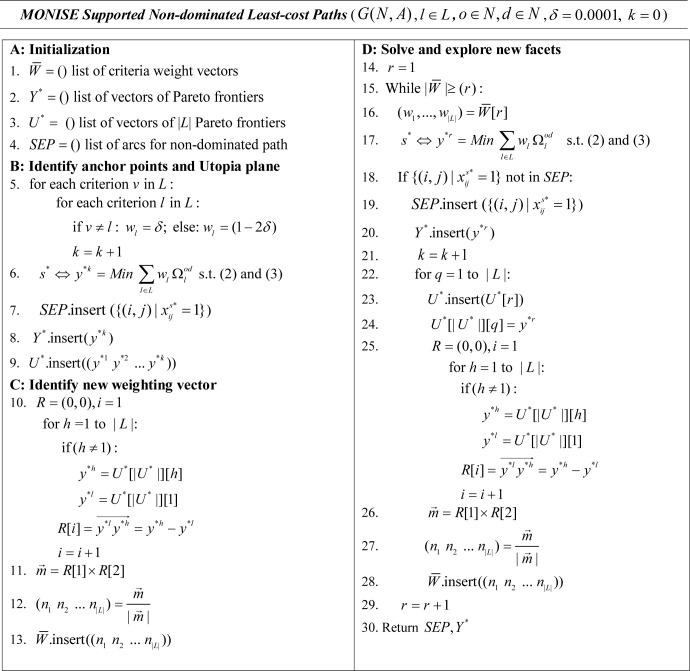
MONISE algorithm for the MOHCP.

To identify the supported efficient solutions for the MOHCP, a MONISE algorithm for identifying supported non-dominated least-cost paths is now outlined in Fig 1. The *MONISE Supported Nondominated Least-cost Paths* algorithm requires a network, attributes for each arc that can be used to measure the objectives (e.g., traversal risk, distance traveled, environmental change) and a pair of origin and destination habitat nodes (*o*,*d*) as input. In Stage A, lists for vectors of objective weights to be applied $\left(\bar{W}\right)$, vectors of the objectives comprising the Pareto frontier for each solution (Y*), vectors tracking sets of Pareto frontiers (U*), as well as storing the arcs comprising the non-dominated paths associated with efficient solutions are initialized. MONISE works by identifying weights for the objectives that will give rise to supported efficient solutions. That is, Objectives (5)–(7) are combined into a single weighted objective (Eq 9) where each weight (*w*_*l*_) has a value [0,1] such that *w*_1_+*w*_2_+*w*_3_ =1.0 as is done in the regular weighting method approach described earlier.

$$Minimize\;w_{1}\Omega_{1}^{od}+w_{2}\Omega_{2}^{od}+w_{3}\Omega_{3}^{od}$$ (9)

In Stage B, a set of initial weights are given to the objectives in (Eq 9) to find the three individual minima (i.e., $Minimize\;\Omega_{1}^{od}$, $Minimize\;\Omega_{2}^{od}$, and $Minimize\;\Omega_{3}^{od}$) known as anchor points [48]. In practice, this equates to applying a large weight to the objective to be optimized and a small, near-zero weight (e.g., *δ* = 0.0001) to the other objectives (step 5) (e.g., *w*_1_ = 0.9998, *w*_2_ = 0.0001, *w*_3_ = 0.0001; in the case of optimizing $\Omega_{1}^{od}$) to ensure the anchor points are not dominated. Objective (9) subject to Constraints (2)-(3) can then be solved (step 6) with the arcs associated with the solution (s*) stored in the list of supported efficient paths *SEP* (step 7) and the Pareto frontier (y*^k^) stored in list Y* (step 8). The Utopia plane defined by the initial three solutions (y*^1^,y*^2^,y*^3^) is then stored in list U* (step 9). In Stage C, the Utopia plane can then be used to derive a new set of objective weights (*n*_1_,*n*_2_,*n*_3_) (steps 10–12) which are stored in list $\bar{W}$ (step 13). Now that a new set of objective weights has been found, they can be used in Stage D in an iterative routine (step 15) to generate and solve a new model (step 16–17). The solution to the new model is then evaluated to see whether or not it has already been found (step 18). If it isn’t present in the set of identified supported efficient paths, it is added to that set (step 19) and its Pareto frontier is recorded (step 20). Next, the Pareto frontier of the new solution is then iteratively swapped into the plane of solutions used to derive the weights used in the model to construct three new planes to add to list U* (steps 23–24). Each of those planes in turn are used to derive three new weighting schemes (steps 26–27) which are added to the list of objective weights to consider (step 28). Any new objective weightings that are found are likewise used to generate and solve additional models (steps 16–17), find new supported efficient solutions (steps 18–20), and generate new weighting schemes to consider until all supported efficient solutions and associated non-dominated paths have been found (step 30). For comparative purposes, the NISE approach for biobjective least-cost paths is outlined in S1 Text.

#### Multi-Criteria labeling algorithm for MOHCP

**Fig 2:**
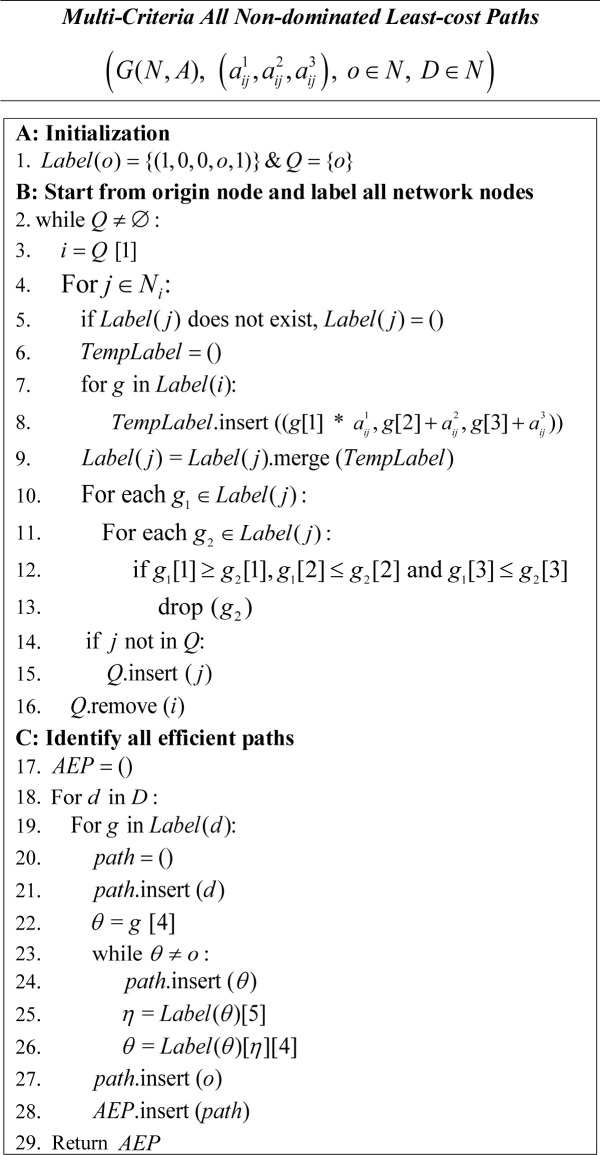
Multi-criteria all non-dominated least-cost paths labeling algorithm for the MOHCP.

The multi-criteria labeling algorithm of Martins [41] can be adapted to accommodate the three objectives in the MOHCP problem to retrieve the full set of efficient solutions (supported and unsupported) from one origin to all destination nodes (Fig 2). The *Multi-criteria All Non-dominated Least-cost Paths* algorithm for MOHCP requires a graph, *G*(*N*,*A*), with arcs attributed with the measures needed to evaluate the objectives $\left(e.g.,a_{ij}^{1},a_{ij}^{2},a_{ij}^{3}\right)$, as well as an origin node and a set of destination nodes. In Stage A, empty list *Q* is initialized that tracks nodes that have been labeled and need to be reconsidered later in the solution procedure. The origin node is then labeled with a set of initial values, a 5-tuple in which the first three elements reflect objective values when traveling from origin node to the labeled node and the last two referencing the index of the preceding node and an id for the label, respectively. These initial values are to assist with computing objective values at the first move when departing the origin node toward an adjacent node. The labeled origin node is then added *Q* (step 1). In Stage B, for each labeled node *i* in *Q* (step 3), the objective values of the neighboring nodes *N*_*i*_ = {*j*|(*i*,*j*)∈*A*} (step 4) are re-computed as accessed through node *i* (steps 5–9), with their labels updated accordingly. A filtering technique is applied to drop dominated paths that may be encountered whenever a set of labels is updated or changed (steps 10–13). Whenever a new node (*j*∈*N*_*i*_) is visited, its label set is evaluated to check if the set of non-dominated paths from origin node to that node have changed or not. Should a node’s label be updated, it is added to *Q* for reconsideration (steps 14–15). Finally, the incumbent node *i* is removed from *Q* (step 16) and the process continues until all nodes are visited and labeled. In Stage C, the supported and unsupported non-dominated paths are retrieved by tracking labels, from each destination node back to the origin node using the reference index to the predecessor node embedded in each label (steps 18–27) and placed into the list *AEP*.

### Application to amphibian habitat connectivity

The MOHCP is now applied to model paths/corridors that could support amphibian habitat connectivity to illustrate the applicability of the multiobjective optimization framework and solution approaches.

#### Factors affecting amphibian habitat connectivity

The persistence of amphibians depend on aquatic and terrestrial habitat, and the ability to successfully migrate and disperse [49,50]. There is some doubt as to the amphibians’ ability to accurately orient themselves with respect to prospective new habitat [6,51]. However, there is evidence that movements toward and away from breeding sites are nonrandom. For instance, Walston and Mullin [52] report that the initial orientation of juveniles from breeding ponds may be influenced by the width of surrounding forested habitat. There is an increasing body of research that has noted the effects that different types of landscape conditions may have on the ability of amphibians to traverse the landscape. For example, Lowe et al. [26] report slope between habitat having a negative effect on gene flow and dispersal. In another study, Giordano, Ridenhour, and Storfer [27] report limited gene flow between high-altitude and low-altitude sites, highlighting the negative impact of elevation change on dispersal. Amphibian movement is known to be influenced by changes in moisture conditions, perhaps in attempts to minimize risk of desiccation and depredation [25]. Traversal distance, the total distance covered in moving from one habitat to another, has also been reported as a factor affecting the movement of amphibians [53] and is viewed as an important factor when modeling cost and likelihood of successful dispersal over the landscape [24]. Therefore, three objectives that may be relevant to amphibian habitat connectivity that fit into the general MOHCP framework are: a) minimize traversal risks associated with land use/land cover types, b) minimize distance and deviation from ideal moisture conditions, and c) minimize change in elevation, which relate to Objectives (5)-(7) in the MOHCP respectively.

#### Study area and experimental design

**Fig 3:**
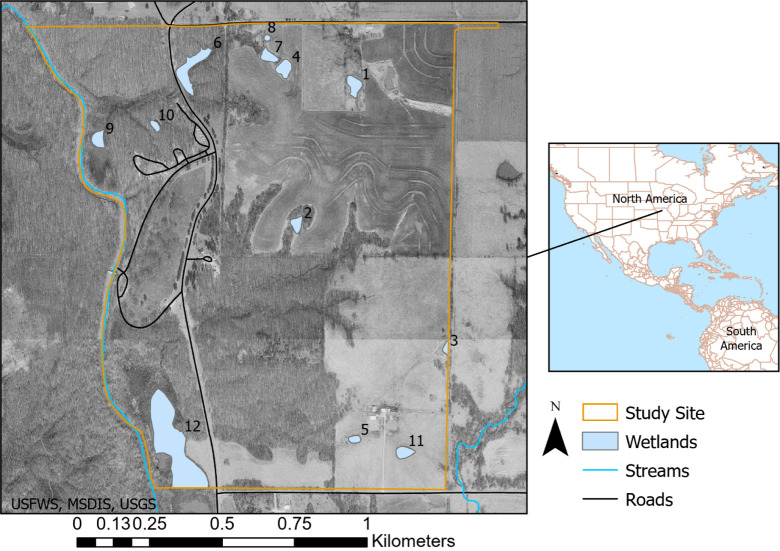
Study site.

The MOHCP and solution methodologies outlined earlier are applied to model landscape paths supporting amphibian habitat connectivity in a portion of Pershing State Park, located in the state of Missouri, USA (Fig 3). This area hosts a variety of wetland types and other landscape features including woody-dominated wetland, deciduous forest, deciduous woody, grassland, cropland, open water and impervious surface [54]. The study site contains 12 wetlands which are considered to be viable origin and destination amphibian habitats.

**Fig 4:**
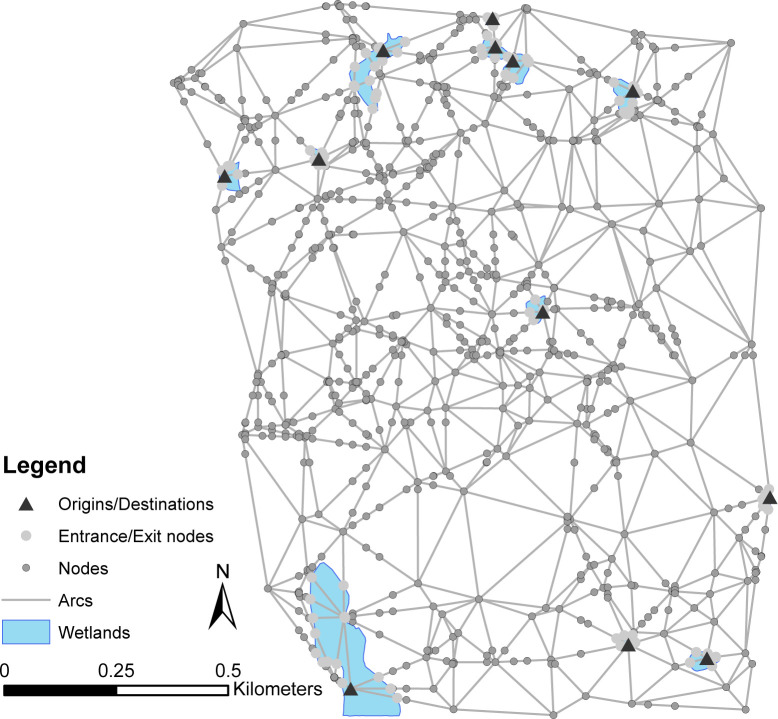
Network representation of wetland system.

While many studies of habitat connectivity utilize a raster-based model of the landscape as a basis for the network, vector-based models can be used as well [55], especially when the landscape characteristics exhibit homogeneity over larger areas as is the case with the current study site. Wetland polygons [56] were used to represent amphibian habitat within the region. To represent the landscape to be traversed, each wetland polygon was rendered as a network node. The areas intervening the wetlands were also rendered as nodes located to represent the spatial variation in land use/ land cover in the region and arcs were added between neighboring nodes. Nodes were then added at locations where the network arcs intersected land use/ land cover polygon boundaries to ensure each arc only traverses one land use/ land cover category. A total of 909 nodes and 1,277 arcs were involved in the resulting network representation of this system (Fig 4).

**Fig 5:**
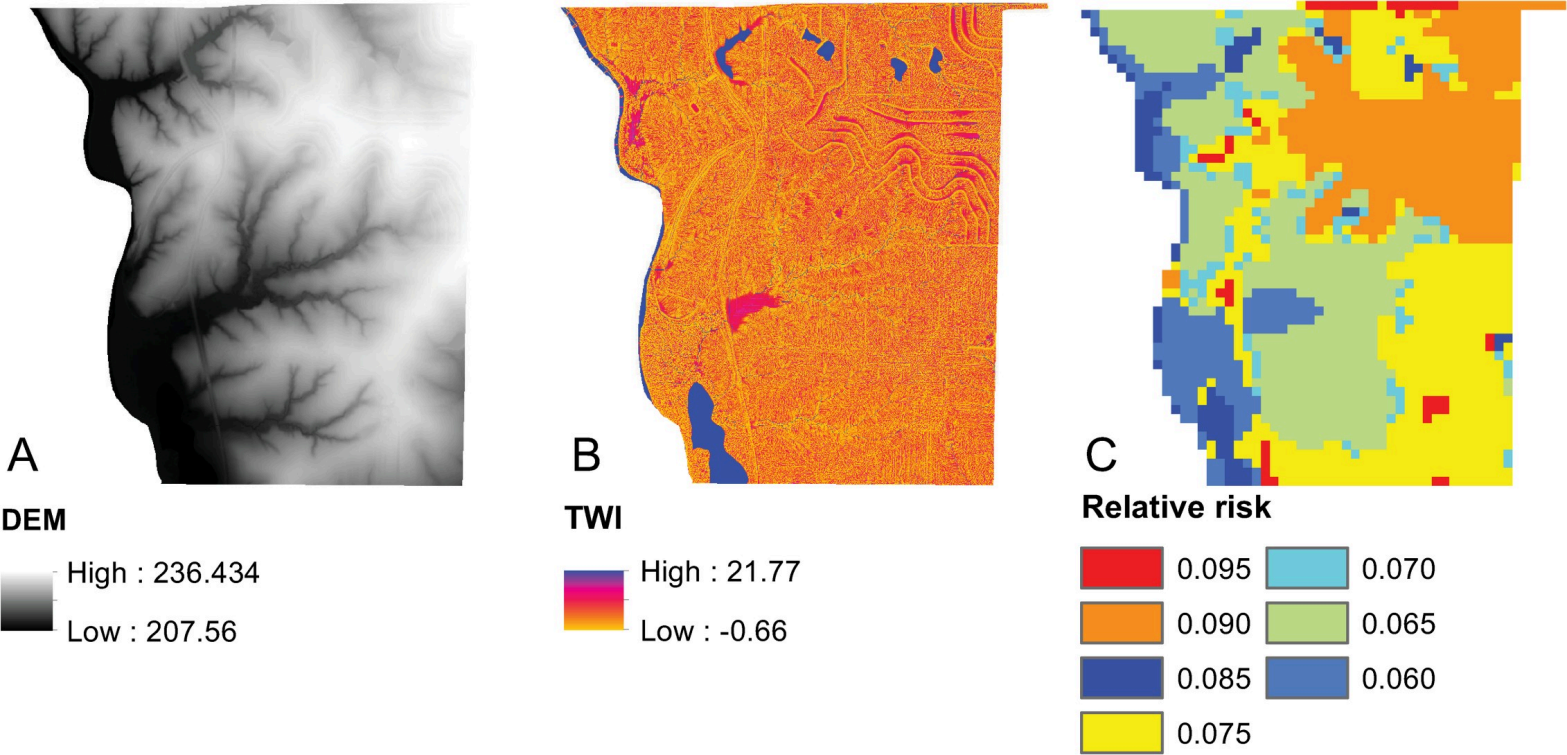
Elevation, TWI, and relative risk.

The arc attributes needed to assess Objectives (5), (6) and (7) were then derived from supplementary layers of geographic data. The elevation of each node was extracted from a digital elevation model (DEM) [54] (Fig 5A). The effects of elevation change were calculated for each arc by subtracting elevation of the end nodes *e*_*j*_ from that of starting nodes *e*_*i*_. Elevation change was classified as either uphill or downhill where uphill movements were weighted twice as high as downhill movements based on their perceived negative impact to movement as in Eq (10) to compute *z*_*ij*_.

$$z_{ij}=\left\{ \begin{array}{l} w_{p}(e_{j}-e_{i}),\;\mathrm{if}\;e_{j}\geq e_{i}\\ w_{n}(e_{i}-e_{j}),\;\mathrm{if}\;e_{j}<e_{i} \end{array}\right.\;\forall (i,j)\in A$$ (10)

Surface moisture was estimated using the topographic wetness index (TWI) index of Beven and Kirkby [57]. The TWI is formulated as *TWI* = ln(*α*/tan(*β*)), where *α* is the drainage area and *β* is local slope. Drainage area (*α*) and local slope (*β*) were derived from the DEM. When calculating the TWI index, locations having zero slope and a non-zero drainage area were given the maximum meaningful TWI value (*TWI* = 21.77) over the study area, and locations having zero slope and zero drainage area were given the lowest meaningful value (*TWI* = −0.66). *TWI* for the study region is shown in Fig 5B. The cost weighted deviation of the soil moisture (*m*_*ij*_) along an arc (as measured using *TWI*) from ideal surface moisture conditions for amphibians (*M*) was then computed as ((*M*−*m*_*ij*_+1)*c*_*ij*_). That is, when soil moisture is low relative to the ideal level, the greater the deviation and associated cost to traversal. Land use/land cover was used as a basis for characterizing traversal risk (Fig 5C). First, each arc was associated with its underlying land use/ land cover [54]. Land use/land cover categories were assigned a base level of risk (*π*^*b*^) (Table 1). Since longer arcs pose higher exposure to a risk category, an adjustment function was applied (*π*_*ij*_ = *π*^*b*^+*c*_*ij*_*π*^*b*^/2*c*_max_) such that the base risk level is increased up to 50.0% based on the length of an arc (*c*_*ij*_) relative to the longest arc (*c*_max_) in the network. Finally, arcs within wetland polygons were attributed with zero costs given that characteristics within each wetland were assumed to be homogenous. Should significant variations exist within a habitat area, the habitat would best be represented as multiple polygons/nodes. A summary of the arc attributes used to represent the three objectives is provided in Table 2 and the complete network dataset can be accessed at: https://doi.org/10.6084/m9.figshare.12609404.v1.

**Table 1:** Relative risk associated with traversal of categories of land use/land cover.

Land use/land cover class	Relative risk (*π*^*b*^)	Area (sq. km)
Woody-dominated wetland	0.060	0.183
Deciduous forest	0.065	0.531
Deciduous woody/Herbaceous	0.070	0.095
Grassland	0.075	0.572
Open water (river)	0.085	0.085
Cropland	0.090	0.428
Impervious surface	0.095	0.035
		Total = 1.929

**Table 2:** Summary of arc attributes.

Variable	Mean	SD	Min	Max
*π*_ij_*	0.080	0.012	0.060	0.135
*m*_ij_**	3.31	3.77	- 0.66	21.77
*z*_ij_***	1.645	1.906	0.0	13.293
*c*_ij_***	49.879	49.043	0.006	305.195

* % likelihood

** *TWI*

*** meters.

## Results and discussion

### Solving the MOHCP

Both the *MONISE Supported Non-dominated Least-cost Paths* algorithm and the *Multi-Criteria All Non-dominated Least-cost Paths* algorithm were applied to solve the MOHCP for the landscape network representing prospects for amphibian movement in the study site. The algorithms were implemented using Python 3.6.6 on a Windows 10 64-bit with five 1.80 GHz processors and 16.0 GB RAM. The optimization solver Gurobi 9.0 was used to find the optimal solution to weighted models in the MONISE routine (steps 6 and 17 in Fig 1). Example implementations of these algorithms can be accessed at: https://doi.org/10.6084/m9.figshare.12609404.v1.

The *MONISE Supported Non-dominated Least-cost Paths* algorithm was executed 132 times, once for each origin-destination pair, identifying all 620 supported efficient solutions in 13.40 minutes. The *Multi-Criteria All Non-dominated Least-cost Paths* routine was executed 12 times, once for each origin, identifying all 3,550 efficient solutions and associated non-dominated paths (supported and unsupported) in 34.46 minutes (solutions can be accessed at: https://doi.org/10.6084/m9.figshare.12609404.v1). Therefore, it is easy to see that the unsupported paths constitute more than 82% of the non-dominated paths, paths that would be ignored in other estimation procedures such as the weighting method and MONISE. For individual origin-destination pairs of wetlands, the number of supported non-dominated paths range from 1–25, while the number of all non-dominated paths (both supported and unsupported) range between 1–183. One explanation for the relatively high proportion of unsupported non-dominated paths is that in even networks of moderate size, a wide variety of diverse paths can exist and hence, there are many complex tradeoffs among the objectives that can manifest.

### Solution characteristics

The number of non-dominated paths originating from and destined to each wetland are reported in Table 3. In general, wetlands with a larger number of supported non-dominated paths also tend to have a larger number of unsupported non-dominated paths. The number of arcs entering each wetland vary based on their size, shape, and relationship with other land use/ land cover areas. The smallest wetland (perimeter = 70.8 m) has only three entrance/exit nodes while the largest wetland (perimeter = 957.2 m) has 14 entrance/exit nodes. As such, some wetlands are going to have more prospective paths given that more opportunities for entrance/exit may exist.

**Table 3:** Number of supported and unsupported non-dominated paths identified for each wetland.

Wetland ID	Perimeter (m)	# entrance/exit nodes	# supported		# unsupported		# all	
			Incoming	Outgoing	Incoming	Outgoing	Incoming	Outgoing
1	214.8	9	34	27	139	128	173	155
2	166.5	6	43	43	154	122	197	165
3	117.4	5	43	41	163	162	206	203
4	194.4	8	52	48	158	166	210	214
5	113.7	8	54	56	204	173	258	229
6	524.1	9	50	46	194	204	244	250
7	181.9	7	32	34	203	250	235	284
8	70.8	3	38	35	216	257	254	292
9	190.3	5	45	50	262	280	307	330
10	116.2	6	67	84	305	263	372	347
11	172.8	5	74	70	293	296	367	366
12	957.2	14	88	86	639	629	727	715
Sum	3020.1	85	620	620	2930	2930	3550	3550

For supported non-dominated paths, the average objective values with respect to likelihood of successful traversal, deviation from ideal soil moisture weighted distance (cost and moisture level shown separately), and elevation change are detailed in Table 4. In aggregate, the supported non-dominated paths tend to have better average objective values with respect to all modeled objectives than the unsupported paths. One reason for this is that there are many more unsupported paths between distant wetlands given more diverse opportunities for routing exist. As discussed earlier, the supported non-dominated paths are only a subset of the full non-dominated set. While the computational time required to identify the supported set using the MONISE algorithm is approximately 37% of that needed to identify the complete set of non-dominated paths, the supported non-dominated solutions only constitute 17% of the full set of non-dominated paths (supported and unsupported). Considering the smaller size of supported non-dominated set and larger standard deviation among the routing objectives in those solutions, it is clear that analysis and decision-making based upon only consideration of the supported efficient solutions (or a subset thereof) is rather limiting given those solutions represent such a small proportion of the efficient set.

**Table 4:** Summary of movement objectives for supported and unsupported non-dominated paths.

Path attribute	Supported non-dominated paths			
	Mean	SD	Min	Max
1−*π*_ij_*	0.24	0.16	0.02	0.91
*m*_ij_**	23404.05	9455.75	1096.88	46028.09
*z*_ij_***	42.74	21.91	0.52	110.42
*c*_ij_***	1329.47	551.45	50.31	2401.98
	**Unsupported non-dominated paths**			
1−*π*_ij_*	0.14	0.09	0.02	0.70
*m*_ij_**	29215.34	7585.58	5318.42	47667.66
*z*_ij_***	53.47	19.86	5.25	115.59
*c*_ij_***	1641.89	443.12	278.58	2693.71

* % likelihood

** *TWI*

*** meters

**Fig 6:**
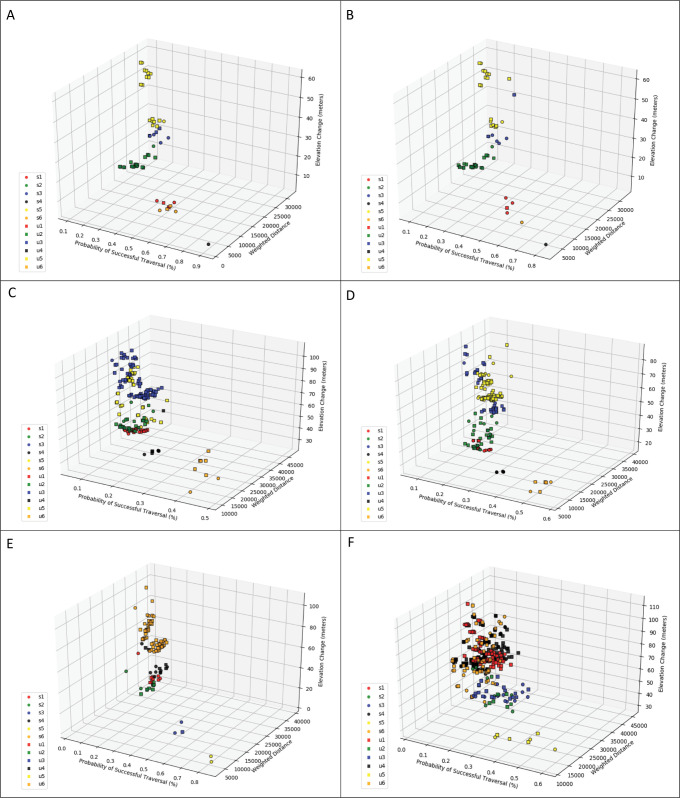
The Pareto frontier for paths destined to wetlands 1, 2, 3, 4, 5, and 6 from: A) wetland 7, B) wetland 8, C) wetland 9, D) wetland 10, E) wetland 11, and F) wetland 12.

Each panel in Fig 6 depicts the Pareto frontier for paths from one origin wetland to six of the destination wetlands (wetland ids correspond with those in Fig 3). The circles represent supported non-dominated paths while the squares represent unsupported non-dominated paths. For example, Fig 6A shows the frontier for paths originating at wetland 7 destined to wetlands 1 through 6. There is only one non-dominated path (which is a supported path) between wetland 7 and 4 and it has the lowest weighted distance, lowest elevation change, and highest probability of successful traversal. That is reasonable given that the wetlands are extremely close together. Wetland 7 is a little further from wetlands 6 and 1 and there are two supported and two unsupported non-dominated paths connecting it to both. Again, without using the label correcting approach, 50% of the non-dominated paths would have been missed. In cases in which wetlands are separated by greater distance and more diverse network structure, options for movement can exhibit much more variation. For example, wetlands 7 and 5 are both relatively small and far apart. However, there are many more non-dominated paths, 3 of which are supported with the other 15 being unsupported. All of these paths have relatively low probabilities of traversal success (0.11–0.21%), but have quite a bit of variation in elevation change (24.6–59.8m) and a small amount of variation in their weighted distance (25,391–30,962). Fig 6F shows the frontier for paths originating at wetland 12 destined to wetlands 1 through 6. Wetland 12 is relatively large and has multiple entrance/exit nodes. As such, there are more opportunities for finding competitive combinations of objectives. A majority of the non-dominated paths in this case are unsupported and the diverse nature of the tradeoffs between the objectives can be seen. Consider for instance the frontier for paths between wetlands 12 and 1. In this case, there are three supported and 79 unsupported non-dominated paths. So again, if one were to only identify the supported non-dominated paths in this example, more than 96% of the other non-dominated paths would be ignored. Among these paths, the probability of successful traversal ranges from 0.02–0.19%, with elevation change ranging from 50.4 to 111.2m and weighted distance ranging from 28,790–44,815.

**Fig 7:**
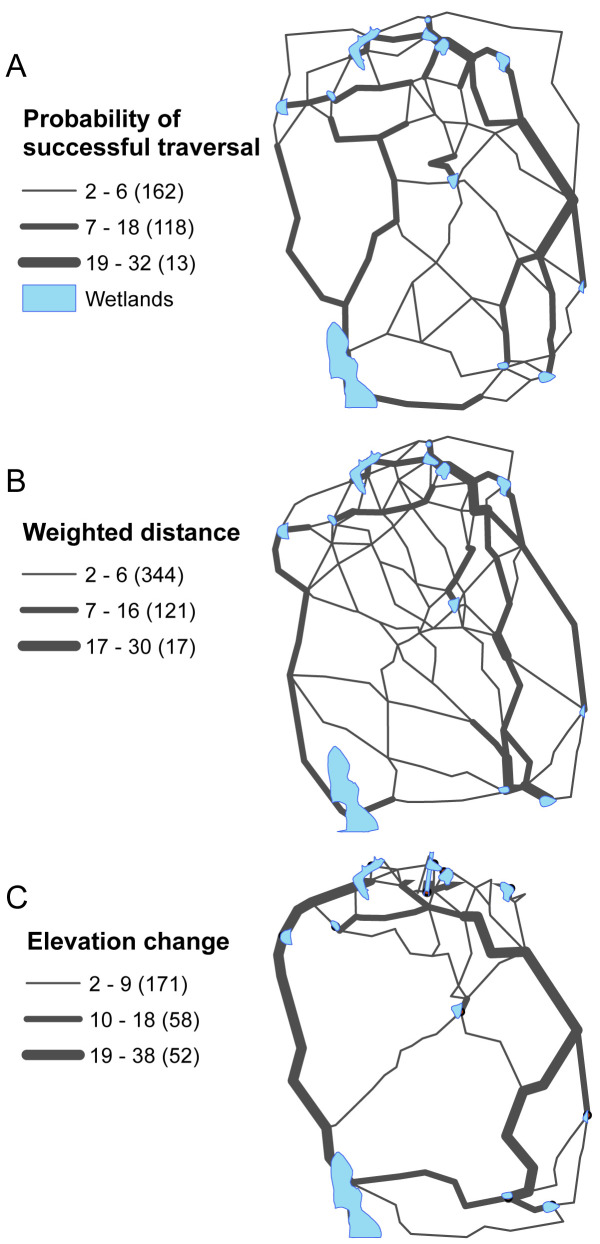
Number of non-dominated paths using arcs in anchor solutions with respect to A) Ω_1_ (probability of successful traversal), B) Ω_2_ (moisture weighed distance), C) Ω_3_ (elevation change).

Fig 7 classifies each arc by the number of supported non-dominated paths traversing it in the anchor point solutions (those optimizing each individual objective as in steps 5–6 in Fig 1). In this sense, there are 132 non-dominated paths for each objective (one path between each pair of wetlands). When optimizing the probability of successful traversal (Fig 7A) only 293 of the 1,277 network arcs (22.9%) are traversed by a non-dominated path. The majority of those (162) are traversed by 6 or less paths with only 13 being traversed by 19 or more paths. When optimizing weighted distance (Fig 7B) 37.7% of the network arcs are traversed by a non-dominated path, indicating that more arcs are favorable in some way toward that objective. A majority of those (344) are still traversed by 6 or less paths. Fig 7C shows the non-dominated paths resulting from optimizing the elevation change objective. In this case, only 22% of the arcs are traversed by a path and there are more arcs (52) that are traversed by 19 or more paths indicating greater consolidation of utility among the wetlands. It should be noted that for any of the three objectives (Fig 7A, 7B and 7C), there are instances in which arcs traversed by non-dominated paths according to that objective are not utilized at all by paths non-dominated with respect to one or both of the other objectives.

**Fig 8:**
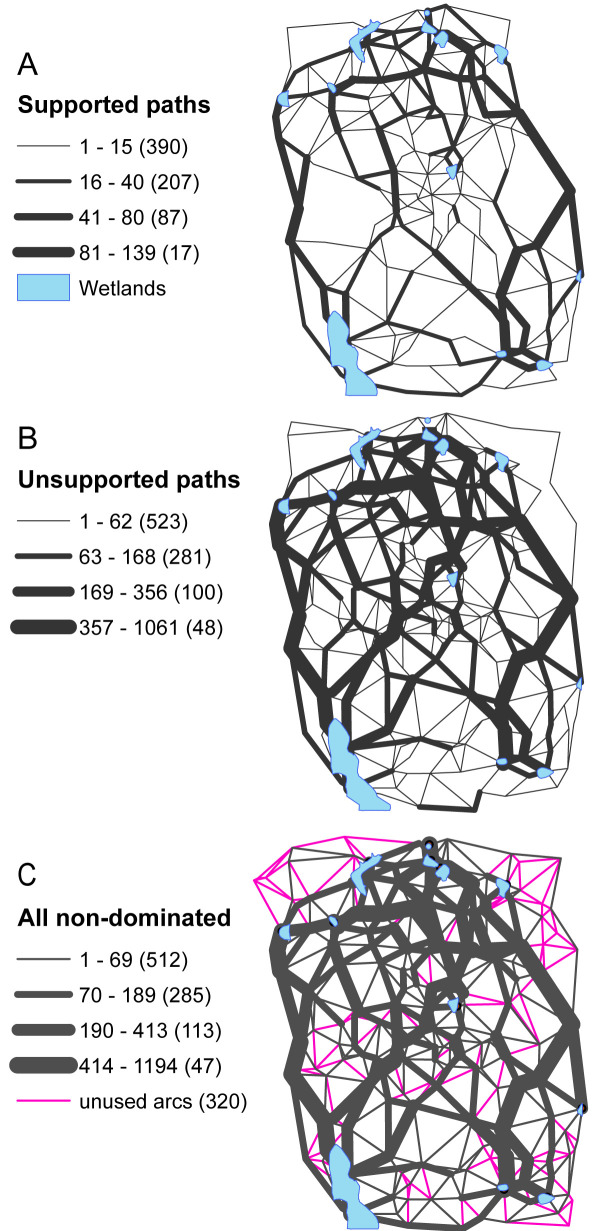
Non-dominated paths: A) supported, B) unsupported, and C) all.

The spatial distribution of the supported and unsupported non-dominated paths is shown in Fig 8. Fig 8A shows the number of supported paths that traversed each arc. In this case, approximately 55% of the arcs are traversed by at least one supported path (unused arcs are not shown). There are clearly some portions of the network that are much more utilized than others. Fig 8B shows the number of unsupported paths traversing each arc. These unsupported paths traverse approximately 75% of the arcs in the network, making use of 20% more of the system than the supported paths. Many of the arcs that were heavily traversed by supported paths are also heavily traversed by unsupported paths, emphasizing their role in the system. However, there are also some arcs that were used to a lesser extent by the supported paths that are used much more by the unsupported paths. For some additional perspective, Fig 8C shows the spatial distribution of all the non-dominated paths (supported and unsupported) as well as the arcs that are never traversed by a non-dominated path. These unused arcs account for 25% of the network arcs, many of which occur near the periphery of the wetland system.

## Conclusions

Assessing prospects for habitat connectivity involves consideration of a complex mixture of factors. A common approach in this respect is to construct a multiobjective least-cost path model to reason about prospects for habitat connectivity. A major realization of many studies is that while multiobjective least-cost paths do allow for the tradeoffs among unique combinations of movement criteria to be evaluated, sometimes even minor changes in how the criteria are combined can give rise to completely different solutions and interpretations of a landscape. One reason for this is that multiobjective least-cost path problems may indeed have a tremendous number of solutions that are best in some respect, known as Pareto optimal or efficient solutions. There are two general categories of Pareto optimal solutions, known as supported and unsupported efficient solutions. Most commonly used solution techniques for multiobjective least-cost path problems can identify the supported efficient solutions (or a portion thereof). However, the number of supported efficient solutions can be very small relative to the number of unsupported efficient solutions. Unfortunately though, most applications of multiobjective least-cost path models only identify a very small proportion of the supported efficient solutions given the solution methodologies that are typically employed. As a result, the solutions that are used as a basis for analysis may only serve as a weak estimate of the connectivity that may actually exist.

To address these issues, this article first provides an overview of the least-cost path problem in the context of ecological research, the distinction between supported and unsupported efficient solutions to least-cost path problems, and methods that can be used to identify each. Next, a multiobjective least-cost path model that accounts for a general set of objectives that are thought in some way to influence movement: a) minimizing risk, b) minimizing distance, and c) minimizing change is formally described. Deriving solutions to a three objective model such as this can be very challenging and as such, two alternative methods for deriving efficient solutions to the model are detailed. The first solution method is a multiobjective non-inferior set estimation (MONISE) algorithm for identifying all supported efficient solutions and associated non-dominated least-cost paths. While the MONISE approach can identify the supported efficient solutions, it cannot identify the unsupported efficient solutions. As such, a multi-criteria least-cost path labeling algorithm is extended to identify all efficient solutions (supported and unsupported) to the multiobjective least-cost path model.

The developed multiobjective least-cost path model is then applied to evaluate prospects for amphibian habitat connectivity in a wetland system to demonstrate the approach. In such applications, the weighting method is typically used to integrate the modeling objectives, resulting in the identification of a handful of supported efficient solutions. However, to illustrate the extensive and diverse set of solutions that can exist, the MONISE and multi-criteria labeling algorithms are applied to more rigorously identify efficient solutions to the model. It was found that the MONISE approach can quickly and efficiently identify all the supported efficient solutions to the multiobjective model. The supported efficient solutions on their own, provide only an estimate of the solutions in the efficient set. However, despite being a little more computationally demanding, the multi-criteria labeling approach is able to identify *all* supported efficient solutions as well as *all* unsupported efficient solutions to the model. Of particular note is that 82% of the efficient solutions were in fact unsupported. Therefore, simply focusing on identification and analysis of supported efficient solutions (or small subset therein) could risk overlooking a significant proportion of viable and potentially important alternatives for habitat connectivity. Thus, analyst should be wary of interpretative problems that may arise when basing analysis on a limited sample of the efficient solutions to multiobjective least-cost path problems.

Modeling habitat connectivity is extremely challenging and can be subject to many uncertainties. Reasoning about the exact mixture of factors that underlie movement involves both field research as well as exploratory analysis. Multiobjective modeling approaches allow for the tradeoffs among the wide variety of factors that could influence habitat connectivity to be better evaluated. As shown in this research, in some instances these tradeoffs may be relatively straightforward. However, in others they may be more complex and perhaps not very intuitive. Regardless, providing environmental planners, managers, and decision makers with a complete set of tradeoffs will allow for a better understanding as to how elements of the landscape act to facilitate or impede habitat connectivity.

## Supporting information

S1 Text A NISE algorithm for the biobjective least-cost path problem. (PDF)
