# Supplementary material for: Modeling habitat connectivity in support of multiobjective species movement: An application to amphibian habitat systems
Source: PLoS Comput Biol. 2020 Dec 28;16(12):e1008540. doi: 10.1371/journal.pcbi.1008540 (PMC7793291; doi:10.1371/journal.pcbi.1008540)
Supplement: S1 Text — (PDF) [file pcbi.1008540.s001.pdf]

Supporting information: "Modeling habitat connectivity in support of multiobjective species movement: an application to amphibian habitat systems"

T.C. Matisziw, A. Gholamialam, and K.M. Trauth

(December 2020)

S1 Text. A NISE algorithm for the biobjective least-cost path problem

In cases in which two objectives are to be optimized, the non-inferior set estimation (NISE) method can be applied to identify all supported solutions as an estimate of the efficient set [1]. In the case of least-cost path models, the *NISE Supported Non-dominated Least-cost Paths* algorithm outlined below can be applied. The notation used in the pseudocode and description follows that detailed in the main manuscript.

The *NISE Supported Non-dominated Least-cost Paths* process involves evaluating the solution space between pairs of supported efficient solutions to detect the presence of another supported efficient solution. In Stage B, given Ω_1 and Ω_2, one would first solve for y^{*1} and then y^{*2} in steps 5 and 6. Then, the equation of the line connecting the two identified solutions (Lines 12 and 13 in Stage C) could be used to identify intermediate solutions by weighting the objective function as in step 18 in Stage D. When new supported efficient solutions are found in Step D, the solution space between them and their neighboring supporting solutions are in turn evaluated for the presence of additional supported efficient solutions. This process continues until all regions of the frontier are explored and all supported efficient paths and their objective values are returned in step 32.

NISE Supported Non-dominated Least-cost Paths $(G(N,A), l \in L, o \in N, d \in N, \delta = 0.0001,\ k = 0)$

A: Initialization

$\bar{W} = ()$ list of criteria weight vectors

$Y^* = ()$ list of vectors of Pareto frontiers

$U^* = ()$ list of vectors of $|L|$ Pareto frontiers

$SEP = ()$ list of arcs for non-dominated path

B: Identify two individual minima

for each criterion v in L:

  for each criterion l in L:

    if $v \neq l$: $w_l = \delta$; else: $w_l = (1 - 2\delta)$

    $k = k + 1$

  $s^* \Leftrightarrow y^{*k} = Min\ w_1\Omega_1^{od} + w_2\Omega_2^{od}$

    s.t. (2) and (3)

  $SEP.\text{insert}(\{(i,j) \mid x_{ij}^{s^*} = 1\})$

$Y^*.\text{insert}(y^{*k})$

$U^*.\text{insert}((y^{*1}\ y^{*2}))$

C: Identify new weighting vector

$y^{*1} = U^*[|U^*|][1]$

$y^{*2} = U^*[|U^*|][2]$

$n_1 = -\dfrac{y^{*2}[2] - y^{*1}[2]}{y^{*2}[1] - y^{*1}[1]}$

$n_2 = 1.0$

$\bar{W}.\text{insert}((n_1\ n_2))$

D: Solve and explore new facets

$r = 1$

While $|\bar{W}| \geq (r)$:

  $(w_1, w_2) = \bar{W}[r]$

  $s^* \Leftrightarrow y^{*r} = Min\ w_1\Omega_1^{od} + w_2\Omega_2^{od}$

  s.t. (2) and (3)

  If $\{(i,j) \mid x_{ij}^{s^*} = 1\}$ not in SEP:

    $SEP.\text{insert}(\{(i,j) \mid x_{ij}^{s^*} = 1\})$

    $Y^*.\text{insert}(y^{*r})$

    $k = k + 1$

    for $q = 1$ to $q = 2$:

      $U^*.\text{insert}(U^*[r])$

      $U^*[|U^*|][q] = y^{*r}$

      $y^{*1} = U^*[|U^*|][1]$

      $y^{*2} = U^*[|U^*|][2]$

      $n_1 = -\dfrac{y^{*2}[2] - y^{*1}[2]}{y^{*2}[1] - y^{*1}[1]}$

      $n_2 = 1.0$

      $\bar{W}.\text{insert}((n_1\ n_2))$

  $r = r + 1$

Return SEP, Y^*

References

1. Cohon J, Church R, Sheer D. Generating multiobjective trade-offs: an algorithm for bicriterion problems. Water Resour Res. 1979;15(5):1001–10.
